# Evaluation of a thoracic ultrasound training module for the detection of pneumothorax and pulmonary edema by prehospital physician care providers

**DOI:** 10.1186/1472-6920-9-3

**Published:** 2009-01-12

**Authors:** Vicki E Noble, Lionel Lamhaut, Roberta Capp, Nichole Bosson, Andrew Liteplo, Jean-Sebastian Marx, Pierre Carli

**Affiliations:** 1 Department of Emergency Medicine, Massachusetts General Hospital, 55 Fruit St., Boston, Massachusetts, USA; 2 SAMU de Paris, Hôpital Necker – Enfants Malades, 149 Rue de Sèvres, 75743 Paris Cedex 15, France

## Abstract

**Background:**

While ultrasound (US) has continued to expedite diagnosis and therapy for critical care physicians inside the hospital system, the technology has been slow to diffuse into the pre-hospital system. Given the diagnostic benefits of thoracic ultrasound (TUS), we sought to evaluate image recognition skills for two important TUS applications; the identification of B-lines (used in the US diagnosis of pulmonary edema) and the identification of lung sliding and comet tails (used in the US diagnosis of pneumothorax). In particular we evaluated the impact of a focused training module in a pre-hospital system that utilizes physicians as pre-hospital providers.

**Methods:**

27 Paris Service D'Aide Médicale Urgente (SAMU) physicians at the Hôpital Necker with varying levels of US experience were given two twenty-five image recognition pre-tests; the first test had examples of both normal and pneumothorax lung US and the second had examples of both normal and pulmonary edema lung US. All 27 physicians then underwent the same didactic training modules. A post-test was administered upon completing the training module and results were recorded.

**Results:**

Pre and post-test scores were compared for both the pneumothorax and the pulmonary edema modules. For the pneumothorax module, mean test scores increased from 10.3 +/- 4.1 before the training to 20.1 +/- 3.5 after (p < 0.0001), out of 25 possible points. The standard deviation decreased as well, indicating a collective improvement. For the pulmonary edema module, mean test scores increased from 14.1 +/- 5.2 before the training to 20.9 +/- 2.4 after (p < 0.0001), out of 25 possible points. The standard deviation decreased again by more than half, indicating a collective improvement.

**Conclusion:**

This brief training module resulted in significant improvement of image recognition skills for physicians both with and without previous ultrasound experience. Given that rapid diagnosis of these conditions in the pre-hospital system can change therapy, especially in systems where physicians can integrate this information into treatment decisions, the further diffusion of this technology would seem to be beneficial and deserves further study.

## Background

The diagnostic tools available to pre-hospital providers when faced with a dyspneic patient are limited. To date, diagnosis has largely relied on physical exam findings such as jugular venous distention, auscultation findings using a stethoscope or infared sensors of pulse-oximetry monitoring hemoglobin oxygenation. These findings are often unreliable (lack both sensitivity and specificity) and are even more difficult to identify in noisy or chaotic environments [1-3]. In addition, many patients have clinical histories or disease processes that overlap a variety of diagnoses and so the pre-hospital algorithm for treatment of acute dyspnea has often been broad-based. For example, a trauma patient with unstable vital signs, evidence of chest trauma and decreased breath sounds in one chest field may be needle decompressed in the pre-hospital system to treat a potential tension pneumothorax. However, this commits the patient to tube thorocostomy as the pleura has now been violated. This invasive, painful procedure mandates hospital admission and monitoring. If the pre-hospital physician could rule out pneumothorax and thus eliminate this diagnosis from the differential, patients could undergo more specific resuscitative measures and avoid unnecessary procedures. Another example of this diagnostic difficulty is the patient with both congestive heart failure (CHF) and chronic obstructive pulmonary disease (COPD). In the pre-hospital system, differentiating wheezing secondary to fluid overload from bronchoconstriction can be very difficult. Both patients are hypoxic and yet the treatments (aside from oxygen) are very different. Does the patient need diuretics and vasodilatation to reduce cardiac pre-load or does the patient need beta-agonists to dilate smooth muscle and steroids to decrease inflammation? Often patients receive both treatments to temporize their clinical condition. However, giving patients with decompensated cardiac function a beta-agonist can have serious detrimental effects and worsen underlying dyspnea [4,5].

Recent studies have shown thoracic ultrasound to be useful in the diagnosis of pulmonary edema and pneumothorax [6-12]. The presence of >3 comet tail artifacts, otherwise known as B-lines, in more than 2 zones per lung field has been shown to have a sensitivity of 100% and specificity of 92% for the diagnosis of pulmonary edema in ICU patients [8]. Thoracic ultrasound, when performed by ultrasound trained physicians, is more sensitive and specific than conventional chest radiography for diagnosing pneumothorax [13,14]. The advent of multiple studies showing the diagnostic abilities of thoracic ultrasound in distinguishing between normal lung, pneumothorax, pulmonary edema and COPD has given the pre-hospital community much to celebrate. Now there is a tool that is portable, easy to use, has minimal risk to the patient and to the provider and has impressive test characteristics for these previously difficult-to-make diagnoses. This is particularly true in the European model for pre-hospital care. In this model the physician, present on the ambulance, directs resuscitation, treatment and transport to the appropriate center for definitive care. Having a portable diagnostic tool that can help distinguish a variety of pulmonary disease processes in this kind of pre-hospital system could be extremely valuable given that medical decision making and resource utilization are physician-based and thus treatment pathways are not as algorithmic as they are in paramedic or emergency technician based systems. If thoracic ultrasound could be taught to these physicians easily, the impact on patient care could be significant.

In the spring of 2007, physicians at the Service D'Aide Medicale Urgent (SAMU) at l'Hôpital Necker in Paris, France underwent training in thoracic ultrasound – specifically looking at image recognition skills in the diagnosis of pneumothorax and pulmonary edema. Pre and post- test scores were collected and trainees gave a subjective evaluation of the utility of TUS and the facility of recognizing thoracic ultrasound images.

## Methods

During a 5-week period from April to May 2007, twenty-seven physicians working for the SAMU at l'Hôpital Necker in Paris France were approached for verbal informed consent to undergo lung ultrasound training and to be tested before and after training to assess image interpretation skills retention. Before any instruction was given, all 27 physicians completed a pre-test evaluation for both applications (pulmonary edema and pneumothorax). Each application pre-test consisted of 25 five-second video clips. Each physician was asked to identify the diagnosis (normal lung, pneumothorax or pulmonary edema) shown in each of the 50 video clips.

Following the pre-test, all twenty-seven physicians underwent two training modules in the ultrasound diagnosis of pneumothorax and pulmonary edema. The modules consisted of a one hour didactic lecture for each application (one hour for pneumothorax and one hour for pulmonary edema) reviewing the principles of lung ultrasound and findings diagnostic for pneumothorax and pulmonary edema. The lectures were given by a physician trained in thoracic ultrasound techniques who was part of the study team. After each lecture, all 27 physicians enrolled in the training were shown the same representative teaching video clips of positive and negative scans. None of the representative clips were included in the pre or post test.

Data was also collected on the amount of previous ultrasound experience each of the 27 physicians had prior to the study. Paired t-tests were used to compare the mean score change pre and post training while two-sample t-tests were used to compare the mean change in scores between those with prior experience and those without any prior experience.

This study was approved by the Partners Human Research Committee.

## Results

Of the 27 physicians who participated in the teaching module, 8 physicians (30%) reported previous ultrasound training but only 4 (14%) had more than one year of ultrasound experience. Only 2 (7%) physicians had any previous experience with lung ultrasound and that was specifically in the diagnosis of pneumothorax. 25/27 physicians reported no thoracic ultrasound experience and reported not being able to identify the pleura on thoracic ultrasound. Therefore, while one-third of physicians had some previous ultrasound experience, that experience was limited and certainly there was minimal exposure or experience with thoracic ultrasound scanning techniques or image recognition prior to this training intervention.

Pre and post-test scores were compared for both the pneumothorax and the pulmonary edema modules (see Table 1 and Table 2).

**Table 1:** Scores for Pulmonary Edema (B-line) Recognition Test

	**Pre-training score**	**Post-training score**
**N**	27	27

**Mean**	14.1	20.9

**Standard Deviation**	5.2	2.4

**95% CI – lower limit**	12.0	19.9

**95% CI – upper limit**	16.1	21.8

**Median**	13.0	22.0

**Q1**	11.0	19.0

**Q3**	17.0	22.0

**Table 2:** Scores for Pneumothorax Recognition Test

	**Pre-training score**	**Post-training score**
**N**	27	27

**Mean**	10.3	20.1

**Standard Deviation**	4.1	3.5

**95% CI – lower limit**	8.7	8.7

**95% CI – upper limit**	11.9	21.5

**Median**	10.0	20.0

**Q1**	7.0	19.0

**Q3**	13.0	23.0

For the pneumothorax module, pre-test scores ranged from 4 (out of 25 possible points) to 19 correct (median score 10). Post-test scores ranged from 14 to 24 correct (median score 20). The mean test scores increased from 10.3 (95% CI 8.7 – 11.9) before the training to 20.1 (95% CI 18.7 – 21.5) after the training (p < 0.001). The standard deviation decreased from pre-test to post-test (from 4.1 to 3.5), indicating a collective improvement.

The difference between physicians with previous ultrasound experience and those without was not significant (mean change of 9.75 vs. 9.84, p = 0.97). Physicians with previous ultrasound experience had a pre-test median score of 11 correct and a post-test median score of 21.5 correct while physicians without any previous ultrasound experience had a pre-test median score of 10 correct and a post-test median score of 20 correct suggesting that there was not extensive previous ultrasound experience with thoracic ultrasound and that previous ultrasound training did not impact thoracic ultrasound image recognition.

For the pulmonary edema module, pre-test scores ranged from 4 to 24 (median score 13). Post-test scores ranged from 14 to 24 (median score 22). The mean test scores increased from 14.1 (95% CI 12.0 – 16.1) before the training to 20.9 (95% CI 21.8 – 22.0) after the training (p < 0.001). The standard deviation decreased again by more than half (from 5.2 to 2.4), indicating a collective improvement.

The difference between physicians with previous ultrasound experience and those without was again not significant (mean change of 6.5 vs. 6.9, p = 0.88). Physicians with previous ultrasound experience had a pre-test median score of 12 and a post-test median score 20 correct while physicians without any previous ultrasound experience had a pre-test median score of 13 and a post-test median score of 22 correct.

When asked if thoracic ultrasound could be helpful in their practice all 27 physicians reported that they felt thoracic ultrasound could positively impact their practice. None reported thoracic ultrasound would have no impact or would negatively impact their practice.

## Discussion

To date, ultrasound has found limited use in pre-hospital settings as the skills required for image application and interpretation required more training than was thought to be clinically expeditious for pre-hospital providers. However, there are many applications where ultrasound performed in the prehospital setting could have a beneficial impact – not only on prehospital treatment algorithms but also on changing destination hospitals or in mobilizing treatment teams prior to patient arrival at destination hospitals.

Thoracic ultrasound is uniquely positioned to make a significant impact on pre-hospital care given that ultrasound can diagnose with high accuracy the presence or absence of interstitial fluid and/or pneumothoraces and thus guide treatment and clinical decision making on these two important causes of dyspnea. The sonographic image patterns are relatively simple (yes/no pleural sliding and yes/no B-line presence) and, as demonstrated by this relatively basic teaching module, require minimal time and effort (one didactic one-hour session) for rapid image recognition improvement.

## Conclusion

Thoracic ultrasound image recognition is readily teachable and minimal didactic and image recognition skill sessions are needed before physicians can recognize the key artifacts which lead to the diagnosis of pulmonary edema and pneumothorax.

Follow-up studies are ongoing to determine the practicality of obtaining these images in the field and the potential outcome benefits in patient care.

## Competing interests

The authors declare that they have no competing interests.

## Authors' contributions

VN, LL, RC, NB, JM and PC participated in the design of this study and carried out the design, coordination and test administration. VN, LL, RC, NB and AL drafted the manuscript and VN performed the statistical analysis. All authors read, revised and approved of the final manuscript

## Pre-publication history

The pre-publication history for this paper can be accessed here:


